# Complete Genome Sequences of Mycoplasma anatis, M. anseris, and M. cloacale Type Strains

**DOI:** 10.1128/MRA.00939-18

**Published:** 2018-09-27

**Authors:** Dénes Grózner, Barbara Forró, Kinga Mária Sulyok, Szilvia Marton, Zsuzsa Kreizinger, Krisztián Bányai, Miklós Gyuranecz

**Affiliations:** a Institute for Veterinary Medical Research, Centre for Agricultural Research, Hungarian Academy of Sciences, Budapest, Hungary; b Department of Microbiology and Infectious Diseases, University of Veterinary Medicine, Budapest, Hungary; Loyola University Chicago

## Abstract

Mycoplasma anatis, M. anseris, and M. cloacale are pathogens of waterfowl. Airsacculitis, nervous disease, and reproductive disorders are the main symptoms in the affected flocks. Here, we report the complete genome sequences of the M. anatis (NCTC 10156), M. anseris (ATCC 49234), and M. cloacale (NCTC 10199) type strains.

## Abstract (precis)

Mycoplasma anatis, M. anseris, and M. cloacale are pathogens of waterfowl. Airsacculitis, nervous disease, and reproductive disorders are the main symptoms in the affected flocks.

## ANNOUNCEMENT

Mycoplasma anatis, M. anseris, and M. cloacale are waterfowl-pathogenic bacteria. M. anatis may cause serious nervous symptoms under stress conditions in ducks (1), while airsacculitis, peritonitis, and the increase of embryo lethality were described after experimental inoculation of the pathogen (2). The type strain was isolated from a duck with sinusitis (3). M. anseris causes airsacculitis, peritonitis, and embryo lethality (4) and probably has a role in cloaca and phallus inflammation of ganders (5). The type strain was isolated from a flock with a history of phallus inflammation (6). The M. cloacale type strain was isolated from a turkey (7), but this species could be isolated from ducks and geese as well (8, 9). Egg infertility is the most common symptom caused by this agent in waterfowl (10). All three of these *Mycoplasma* strains can be transmitted vertically (4, 11, 12). The coexistence of waterfowl-pathogenic mycoplasmas has been described (13).

The M. anatis (NCTC 10156), M. anseris (ATCC 49234), and M. cloacale (NCTC 10199) type strains were purchased directly from the repositories. Cells were grown in Oxoid *Mycoplasma* broth medium (pH 7.8) (Thermo Fisher Scientific, Inc., Waltham, MA) supplemented with 0.5% (wt/vol) sodium pyruvate, 0.5% (wt/vol) glucose, and 0.005% (wt/vol) phenol red and were incubated at 37°C. DNA was extracted with the QIAamp DNA minikit (Qiagen, Inc., Hilden, Germany). DNA libraries were prepared with the Nextera mate pair library preparation kit (Illumina, Inc., San Diego, CA). Two genome sequencing runs were performed on an Illumina NextSeq 500 instrument for each strain, generating 2 × 150-bp (300 cycles) and 2 × 75-bp (150 cycles) mate pair reads. NxTrimm software (14) was used to trim the junction adapters from all the raw mate pair reads, generating shorter paired-end reads as well. First, contigs were generated per strain from the paired-end output data by the SPAdes Genome Assembler 3.11 (15) with the assembly-only option. Then, the paired-end contigs and the trimmed mate pair output data were assembled with the same option, generating the draft genomes. Trimmed reads (mate pair and paired-end) were control mapped to the draft *de novo* genome and curated with Geneious 9.1.8 software (16). Circularization of the contigs was performed by primer pairs and PCR assays specific for the contigs’ ends (data not shown), and the PCR products were sequenced on the ABI Prism 3100 (Applied Biosystems, Foster City, CA) automated DNA sequencer. The NCBI Prokaryotic Genome Annotation Pipeline (17) online service was used to annotate the genomes. The rRNA and the tRNA genes and the transfer-messenger RNA (tmRNA) genes were verified by RNAmmer (18) and ARAGORN (19), respectively.

The total genome sizes and information concerning the strains are detailed in Table 1. We hope that the presented complete and circularized genomes will improve research of the waterfowl-pathogenic *Mycoplasma* species.

**TABLE 1:** Genome information and GenBank accession numbers of *Mycoplasma anatis*, *M. anseris*, and *M. cloacale* type strains^*a*^

Strain	GenBank accession no.	SRA no.	Size (bp)	Coverage (×)	G+C content (%)	No. of CDS^*b*^	No. of rRNAs	No. of tRNAs	No. of ncRNAs^*c*^
M. anatis (NCTC 10156)	CP030141	SRP155810	956,093	292	26.7	791	6	33	1
M. anseris (ATCC 49234)	CP030140	SRP155813	750,010	1,833	26.4	617	6	32	2
M. cloacale (NCTC 10199)	CP030103	SRP155814	659,552	1,439	27.0	541	4	31	2

a The number of tmRNAs was 1 and the number of regulatory elements was 1 for each strain listed.

b CDS, coding sequences.

c ncRNAs, noncoding RNAs.

### Data availability.

The annotated genome sequences were deposited in GenBank, and the raw read data are available in the Sequence Read Archive. The accession numbers are listed in Table 1.
